# Thermal Properties of Seed Cake Biomasses and Their Valorisation by Torrefaction

**DOI:** 10.3390/polym16202872

**Published:** 2024-10-11

**Authors:** Elena Butnaru, Elena Stoleru, Daniela Ioniță, Mihai Brebu

**Affiliations:** “Petru Poni” Institute of Macromolecular Chemistry, 41A Gr. Ghica Voda Alley, 700487 Iași, Romania; elena.butnaru@icmpp.ro (E.B.); elena.paslaru@icmpp.ro (E.S.); ionita.daniela@icmpp.ro (D.I.)

**Keywords:** thermogravimetry, thermal stability, energy yield, biochars, bio-oils

## Abstract

Seed cakes, by-products from the cold press extraction of vegetable oils, are valuable animal feed supplements due to their high content of proteins, carbohydrates, and minerals. However, the presence of anti-nutrients, as well as the rancidification and development of aflatoxins, can impede their intended use, requiring alternative treatment and valorisation methods. Thermal treatment as a procedure for the conversion of seed cakes from walnuts, hemp, pumpkin, flax, and sunflower into valuable products or energy has been investigated in this paper. Thermogravimetry shows the particular behaviour of seed cakes, with several degradation stages at around 230–280 and 340–390 °C, before and after the typical degradation of cellulose. These are related to the volatilisation of fatty acids, which are either free or bonded as triglycerides, and with the thermal degradation of proteins. Torrefaction at 250 °C produced ~75–82 wt% solids, with high calorific values of 24–26 kJ/g and an energy yield above 90%. The liquid products have a complex composition, with most parts of the compounds partitioning between the aqueous phase (strongly dominant) and the oily one (present in traces). The structural components of seed cakes (hemicelluloses, cellulose, and lignin) produce acetic acid, hydroxy ketones, furans, and phenols. In addition to these, most compounds are nitrogen-containing aromatic compounds from the degradation of protein components, which are highly present in seed cakes.

## 1. Introduction

Biomass, with its complex composition consisting of natural polymers (hemicelluloses, cellulose, and lignin) combined with low-molecular-weight bioactive compounds, is an abundant resource of energy and materials. The development of economic growth is inextricably linked to energy consumption. The European Union’s energy objective is to achieve an increase in the use of energy from renewable sources by 2030, in response to the negative environmental impact brought about by the use of fossil fuels [1]. The utilization of agricultural biomass fulfils the concept of a circular bioeconomy, which emphasizes the recycling and reuse of renewable sources and focuses on minimizing waste generation, replacing non-renewable, fossil-based materials [2].

Seed and oilseed crops are a type of agricultural biomass with versatile use; they are grown for the production of edible seeds or for the oils extracted from them, which are used in human consumption or in biofuel production [3]. The total Romanian oilseed production reached almost 4.2 million metric tons (MMT) in 2021/2022. Romania is the largest sunflower producer among the EU Member States, with the largest sunflower area, according to USDA [4]. Rapeseed and soybean oilseed plants are also common in Romania, compared to flax, hemp, castor, or sesame, which are less popular [5].

The seeds of these plants have a high nutritive and energetic value. Their protein content is usually between 6 and 45 wt%, and their lipid content reaches at least 15 wt%, strongly depending on the oilseed variety. They are also rich in fibres, carbohydrates, vitamins, water, and minerals [6]. Oilseed cakes and meals are the by-products left after removing the majority of the oil from the oilseeds, generally accounting for about 50% of the original total seed weight. Seed cakes are the remaining product from the mechanical press method, while meals are the resulting materials after oil extraction using organic solvents such as hexane, xylene, or toluene. The mechanical pressing method is preferred because the process is cheap and rapid and only simple equipment is needed; therefore, it can be used locally on the farm [7].

Oilseed cakes are usually air-dried to remove the water before storage. The stability of oilseed cakes over time, in order to prevent moulding, is dependent on moisture control; a humidity level below 12% is considered safe for storage [8]. Seed cakes are mainly composed of high amounts of fibres [9] and proteins, and around 10 wt% residual oil [10].

Oilseed cakes are generally used as animal feed supplements, especially for ruminants and fish, based on their rich protein, carbohydrate, and mineral content [11]. Hemp and sunflower seed cakes are particularly rich in proteins and fibres. For example, hemp seed cakes contain more than 50 wt% proteins and about 15 wt% fats [12,13], while sunflower seed cakes contain about 30–50 wt% proteins [14], of which helianthinin and albumins are the main ones [15]. As for their fibre content, this varies between ~39 and 56 wt% and between ~35 and 48 wt% in hemp and sunflower, respectively [16]. Flax seed cakes were found to be rich in nutrients such as alpha-linolenic acid (~59 wt%), dietary fibres, proteins, and minerals, of which Ca, Mg, P, and K are the main ones [17]. Pumpkin seed cake contains important quantities of tryptophan, small amount of lysine and isoleucine, and fatty acids such as oleic and linoleic acid [18]. Walnut seed cakes exhibit interesting nutritional values due to their very high content of phenolic compounds; however, tocopherols and some of the phospholipids are removed during oil extraction [19].

Despite their nutritional properties, which make them a preferred source of protein in animal feed, most oilseed cakes also contain significant amounts of anti-nutrient compounds that limit their use for consumption. Specifically, flax seed oil cakes contain cyanogenic glycosides such as linustatin, linamarin, and lotaustralin, which can lead to health complications via hydrogen cyanide poisoning [20]. Hemp seeds contain phytic acid, cyanogenic glycosides, and condensed tannins, which promote protein and mineral deficiency [21]. No anti-nutritional compounds are reported in sunflower oilseed cakes, but these have large quantities of phenolic compounds (2–5%)—mostly chlorogenic acid [22]—and the phenolic–protein interactions lead to negative effects, including changes in their organoleptic properties (dark-green colour due to the oxidation of phenolic compounds), strongly limiting their stability and storage life [23]. In addition, the fatty acids that remain after oil extraction, especially the unsaturated ones, are prone to rancidification. This generates undesirable odours and flavours, making seed cakes less attractive for consumption. Moreover, serious concerns are raised by the development of mycotoxin contaminants in oilseed cakes, which are provoked by fungal growth or by attack from rodents or insects. Different types of oilseed cakes such as sunflower, rapeseed and soybean were previously reported as being contaminated with aflatoxins [24]. These aspects can strongly decrease the quality of seed cakes, impeding their usage as feed supplements. In this context, alternatives are needed for the treatment and valorisation of large quantities of seed cakes into valuable materials or energy, which otherwise could be wasted.

In addition to their high protein content, the seed cakes also contain large amounts of cellulose and lignin, especially from the hulls and skins of the seeds. This makes seed cakes potential lignocellulosic materials that are suitable for conversion into useful products through thermal treatments, which involve the breakdown of molecules by heating them at high temperatures in an oxygen-deficient atmosphere. Depending on the temperature range of the process, this is called torrefaction (~200–300 °C), pyrolysis (~350–600 °C), or gasification (above ~800 °C); the dominant product shifts with increasing temperature from biochars to bio-oils and then to gasses. For example, from the thermal treatment of flax seeds, a maximum biochar yield of ~47 wt% is reached at 350 °C, while ~58 wt% bio-oils are produced at 500 °C [25]. Similarly, ~42 wt% biochars with a high heating value (HHV) of 29 kJ/g and ~52 wt% bio-oils with a HHV of ~33 kJ/g are obtained at 400 and 600 °C, respectively, from rapeseed [26].

Pyrolysis is largely used for the thermal conversion of biomass into biochars and bio-oils with complex compositions [27]. Torrefaction is the preferred mild thermal treatment method for the stabilization of biomass through the removal of humidity and unstable components. This avoids biological decay, increases the energy density, improves compressibility, and reduces the variability in the composition of raw materials, which can then be further subjected to pyrolysis.

Prior to the pyrolysis process, a detailed characterization of the biomass is needed because it offers relevant information related to its thermal behaviour and energetic profile [28]. The fixed carbon and lignin content in oilseed cakes predicts the possibility of producing considerable amounts of biochar via pyrolysis. The fixed carbon content was found to be prominent in raspberry seed cake, and pumpkin oil or hemp oil seed cake [29]. Also, the moisture and ash content are important indicators in heat production. Moisture and ash concentrations higher than 10% lower the calorific value of the fuel, because moisture leads to poor heat transfer among biomass particles, and minerals do not participate in combustion [30].

Previous reports mainly address the thermal valorisation of seed cakes from technical plants used for the extraction of oils in the process of biodiesel production. However, reports on the torrefaction of seed cakes are very scarce, especially regarding those from edible species. A Web of Knowledge database search in September 2024 using “torrefaction seed cake” as a keyword delivers only 14 scientific works, all of which are related to technical plants (*Jatropha curcas*, *Karanja*, and *Calophyllum inophyllum*). While the appearance of situations in relation to the valorization of seed cakes from edible species into energy and/or chemicals is preferred, no study dedicated to this subject has yet been reported, to the best of our knowledge. In this context, the present paper fills the gap addressing the torrefaction of seed cakes from edible species, as a valuable alternative for energetic valorisation when reasons such as contamination, alteration, or the presence of a high content of anti-nutrients impede their use as animal feed supplements.

We previously reported on the characterization and thermal valorisation of forestry biomass residues [31,32]. In this paper, we focus on the thermal valorisation of seed cakes from different sources, in an attempt to convert them into valuable products. Correlations are made between the thermal behaviour, the product yield, the fuel quality of the biochar, and the chemical composition of the bio-oils.

## 2. Materials and Methods

### 2.1. Materials

**Figure 1:**
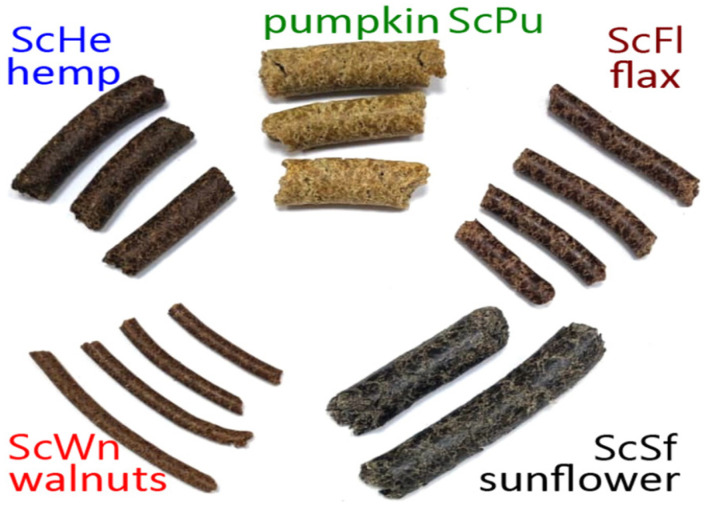
Visual aspects of seed cakes from walnuts, hemp, pumpkin, flax, and sunflower.

Seed cakes of walnuts (ScWn), hemp (ScHe), pumpkin (ScPu), flax (ScFl), and sunflower (ScSf) remaining from the extraction of vegetable oils using cold press procedures were kindly provided by local producers (TAF PRESOIL SRL, 117A Luncani 407362, Cluj, Romania). The colour of the cakes is dependent on the initial raw material variety and its growing conditions. Walnut oilseed cakes are light brown; the colour of pumpkin oilseed cake is yellow to brownish green—Figure 1. Oilseed cake from flax seeds has different shades of brown and appears more heterogeneous, while hemp and sunflower oilseed cakes have a dark brown to black colour. This is in good agreement with other reports [33].

A previous in-lab characterization of seed cakes using specific methods described previously [28] indicates a humidity content of around 8 wt%, with a very narrow variation among samples. The proximate analysis, expressed on a dry basis, shows a volatile matter, and fixed carbon and ash content in the range of 75–79, 14–17, and 5–9 wt%, respectively. The compositional analysis reveals extractives in high amounts (19–26 wt%)—40–48 wt% cellulose, 29–40 wt% lignin, and only very low amounts of 0.3–4.7 wt% hemicelluloses. The ultimate analysis indicates a carbon content ranging between 52 and 65 wt%. The hydrogen content of 5–6 wt % was higher and the oxygen content of 20–36 wt%, was lower than for similar lignocellulosic agriculture biomass waste such as stalks, hulls, shells, and pits. This is due to the high content of fatty acids that still remains in the seed cakes after the cold press extraction of vegetable oils, which are also the main parts of extractives. For example, oleic acid (molecular formula C_18_H_34_O_2_), one of the main components of vegetable oils, contains 12.1 wt% hydrogen and 11.3 wt% oxygen. On the other hand, lignin (general formula C_81_H_92_O_28_) and cellulose (C_6_H_10_O_5_) have only 6.1 and 6.2 wt% hydrogen, while the oxygen content is 29.6 and 49.4 wt%, respectively.

### 2.2. Methods

A Q5000 IR thermogravimetric (TG) analyser from TA Instruments (New Castle, DE, USA) was used to determine the thermal behaviour of the seed cakes. Sample amounts of about 7 mg with a size smaller than 0.1 mm were used for analysis, under a heating rate of 10 °C/min up to 600 °C and an inert atmosphere of nitrogen (25 mL/min flow rate).

**Scheme 1:**
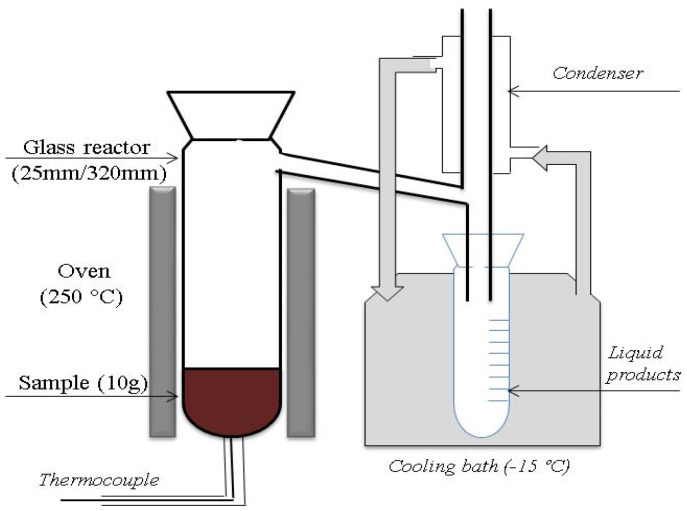
A schematic representation of the torrefaction set-up.

The torrefaction of seed cakes was performed in a semi-batch process, which is schematically presented in Scheme 1, which was detailed in a previous paper [34]. Sample amounts of about 10 g were ground and sieved to dimensions smaller than 5 mm and were placed at the bottom of a glass reactor. The seed cakes were heated with a rate of 10 °C/min up to the final temperature of 250 °C, at which torrefaction proceeds under a self-generated atmosphere. The liquid products were collected after cooling the torrefaction volatiles that left the reactor. The biochar remaining inside the reactor was recovered after the electrical furnace was cooled to room temperature.

A C200 h calorimeter bomb from IKA (Staufen, Germany) was used to determine the calorific value of the seed cakes before and after torrefaction. Samples with particle sizes below 0.5 mm were used, in amounts of ~0.3 g.

A 6890 N gas chromatograph (GC) coupled with a 5975 inert XL mass selective detector (MSD), from Agilent, Santa Clara, CA, USA, was used to determine the chemical composition of the torrefaction liquid products. An HP5-MS column (30 m × 0.25 mm × 0.25 µm) was used for chromatographic separation. The temperature programme was 40 °C (2 min) → 320 °C (10 °C/min). Helium was used as the carrier gas, with a flow rate of 1 mL/min. The inlet and the transfer lines were heated at 230 and 280 °C, respectively, and the samples were injected with a 1:100 split rate. The NIST20 database was used for the qualitative identification of the compounds, imposing a minimum 85% quality of recognition. The Kovats retention index was calculated for each chromatographic peak and was used for the confirmation of identification according to the MS database.

## 3. Results and Discussion

### 3.1. Thermal Behaviour of Seed Cakes

The thermal degradation of biomass reveals a particular behaviour of the structural components, i.e., hemicelluloses, cellulose, and lignin. Lignin decomposes slowly, over a broad temperature range, starting soon after the removal of humidity, extending to above 550 °C when aromatic condensation reactions occur. This appears in the derivative thermogravimetric (DTG) curves as a large and flat peak, most often visible only as a long tail above 400 °C; its first range is covered by the peaks and shoulders of cellulose and hemicelluloses. Cellulose decomposes fast, with a sharp peak at about 300–320 °C in the DTG curves. Hemicelluloses have more thermally labile structures, hence they degrade at lower temperatures, usually appearing as a shoulder at around 240–260 °C before the main peak of cellulose degradation. However, the amount of hemicelluloses in seed cakes is very low; therefore, their clear observation in the DTG curves is not expected. In addition, interactions between the structural components lead to differences in the thermal behaviour of individual components.

**Figure 2:**
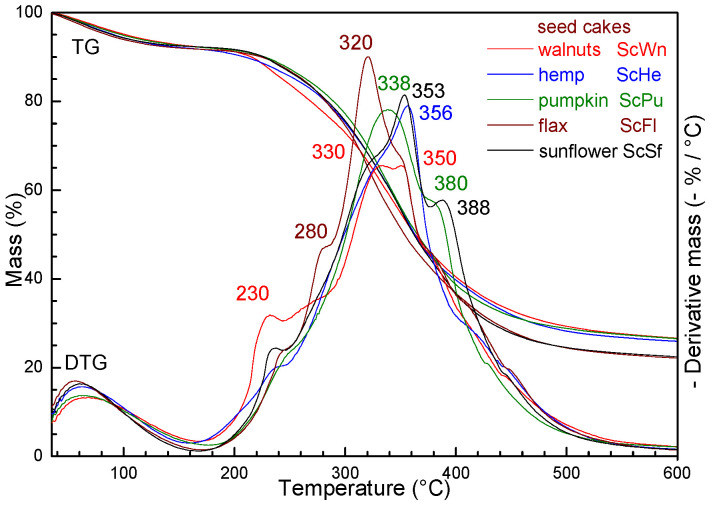
TG/DTG curves of seed cakes from walnuts, hemp, pumpkin, flax, and sunflower.

After the removal of humidity, the thermal degradation of seed cakes occurs in a single step between ~160 and ~520 °C, with a mass loss of ~66–70 wt%; the residual mass at 600 °C is ~22 wt% for flax and sunflower and ~26 wt% for the other three seed cakes—Figure 2. In addition to the main DTG peak at around 320–340 °C, denoting the degradation of cellulose, multiple overlapped stages can be observed in the DTG curves, both below 300 °C and above 350 °C, with each sample showing particular behaviours. This could be due to the complex composition and interactions between components.

Since the studied seed cakes have only very low amounts of hemicelluloses, the shoulders at around 230 °C observed for walnuts, sunflower, and hemp, as well as the shoulder at 280 °C for flax, cannot be related to this structural component. This could be interpreted as volatilisation without the decomposition of the remaining fatty acids in the seed cakes, as has been reported in several studies [35,36], for example, the thermal analysis of long-chain fatty acids, where the volatilisation of palmitic and stearic acid was found to start at around 160–170 °C and end at ~230–240 °C [37]. On the other hand, other researchers [38] have considered that the part of the complex DTG curve observed below 300 °C could be explained by the degradation of the most labile structures in proteins; however, this strongly varies according to the particularities and related interactions of each sample.

It was reported that triglycerides have a maximum volatilisation rate at around 378 °C [39]. Fatty acids can be in free form or bonded as triglycerides, which affects the volatilisation rate, hence the presence of the shoulders in the DTG curves both before and after the peak corresponding to cellulose [40]. Considering the reports on the pyrolysis of microalgal biomass [41], castor oil [42], organic solid waste [43], and proteins and carbohydrates [44], the DTG shoulders at around 380 °C could be interpreted as the advanced stages of protein decomposition [45], especially of the aromatic amino acids [46], which can be affected by interactions. These can explain the DTG shoulders in the 340–390 °C range.

It should be mentioned that the DTG peaks of fatty acids and proteins can be strongly affected by interactions with the polymeric structures in the systems, especially with cellulose and lignin, in which they can be impregnated. This is similar to the proven interactions between vegetal cooking oil and polycarbonates from used CDs and DVDs, which affect thermal co-processing [47]. The thermal degradation of lignin structures in seed cakes can be observed by the small shoulder at around 450 °C in the DTG curve and the long tail following it.

The patterns observed for the DTG curves of the seed cakes confirm the complex and particular composition of the seed cakes and the interaction between the components, with an expected impact on the thermal treatment of these biomass residues.

### 3.2. Torrefaction of Seed Cakes

Torrefaction at 250 °C produces hydrophobic, blackish materials representing 75–82 wt% of the initial seed cakes. The loss of colour indicates changes in chromophore chemical groups, especially the oxygenated ones. These are mainly part of the volatiles and extractives, which are responsible for the initial colour of the materials, but are removed by torrefaction.

**Figure 3:**
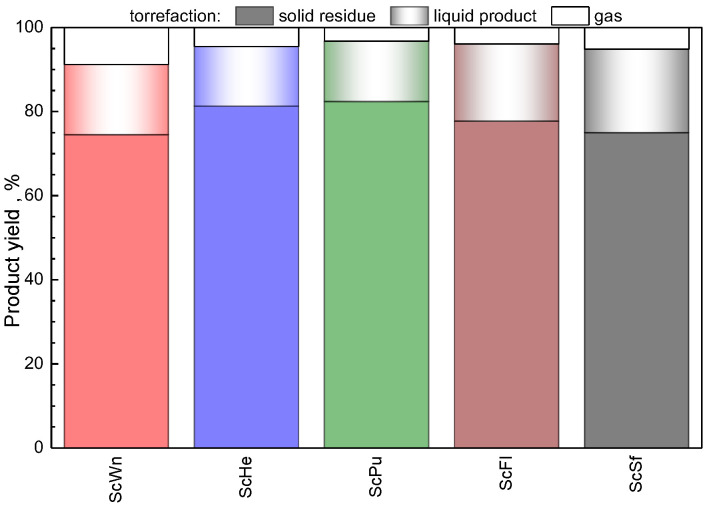
The product yield form the torrefaction of various seed cakes.

The liquid product resulting from torrefaction varies in the ~14–20 wt% range—Figure 3—and mainly consists of an aqueous fraction. In addition to the adsorbed humidity, water also results from the thermal decomposition of labile chemical bonds, especially from the oxygen-containing compounds in lignin, since hemicelluloses are only present in small amounts. The formation of water and carbon dioxide is the main process through which large amounts of oxygen are removed from biomass in the first stages of thermal processing at lower temperatures, and leads to the biological and chemical stabilization of the remaining structures in the torrefied materials. Small amounts of insoluble, dark, brownish droplets of organic oil, with a yield that is difficult to precisely determine, were observed in the liquid product. The organic fraction was dispersed into the aqueous one via vigorous shaking, before gas chromatographic analysis was conducted to determine the contained chemical compounds.

The gas yield was about 3–9 wt%. The seed cakes from walnut and sunflower produced the highest yield of gas and liquid products, while leaving the lowest amounts of torrefied residue. This could be due to their high content of extractives.

**Figure 4:**
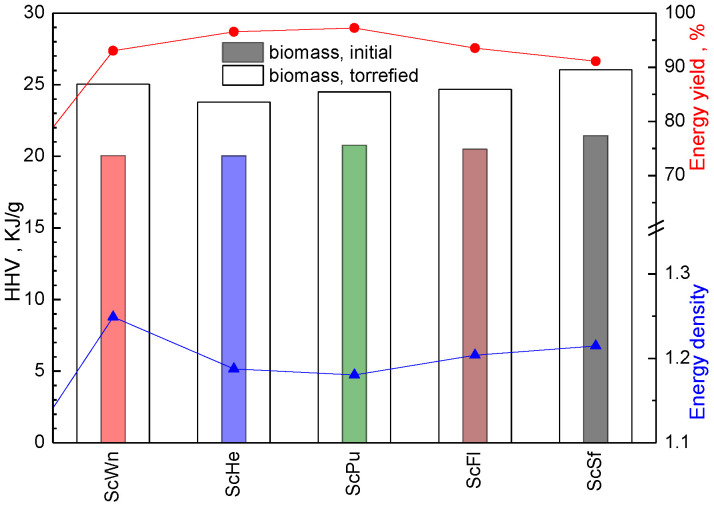
The energetic values (the high heating rate, energy density, and energy yield) from the torrefaction of seed cakes.

Energy efficiency is a very important aspect of thermal treatment processes. Torrefaction increases the calorific value of the solid material from 20 to 21.5 up to 24–26 kJ/g—Figure 4—which brings them close to the category of superior solid fuels, which are useful as a valuable source of energy. This is mainly due to the strongly decreased amounts of oxygen that are removed during torrefaction, mainly as water and carbon dioxide. Indeed, according to Equation (1), oxygen has a strong negative contribution to the theoretical high heating value calculated for solid fuels [48].

HHV = 0.3491 C + 1.1783 H + 0.1005 S − 0.1034 O − 0.0151 N − 0.0211 Ash(1)

Hydrogen has positive contribution to the HHV; however, its amount decreased by torrefaction. Hence, the strong increase in carbon content is the main factor that contributes to the increased calorific value.

Torrefaction increased the energy density of the solid materials to ~1.2, with an energy yield of 91–97 wt%. Most of the thermal energy input required for heating the seed cakes up to a temperature of 250 °C, at which torrefaction was performed, remains stored inside the torrefied materials, while only less than 10% was spent to remove the humidity and the extractives. This makes torrefaction an attractive thermal procedure to convert unused seed cakes into valuable sources of energy.

**Figure 5:**
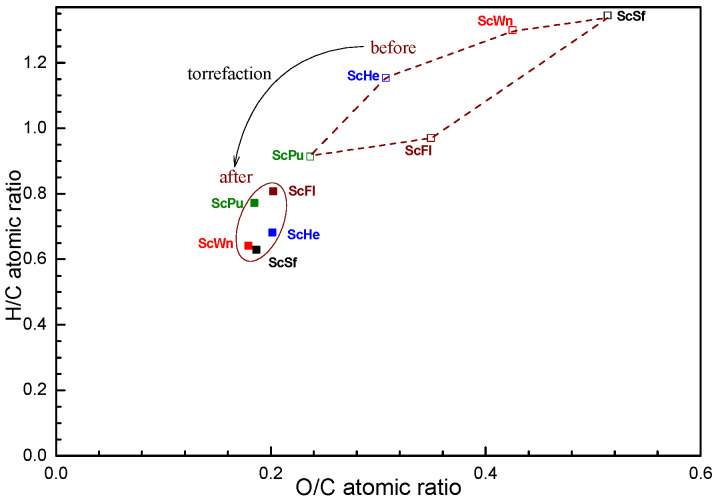
The position of seed cake samples in the Van Krevelen diagram before and after torrefaction at 250 °C.

The above discussed change in the chemical composition of the seed cakes after torrefaction can be better observed from the changed position in the Van Krevelen diagram. This was initially used for a graphical description of kerogen and petroleum and was later extended to other solid fuels. The seed cakes are largely dispersed, with a high variation in the O/C and H/C atomic ratios in the ~0.24–0.5 and ~0.9–1.35 range, respectively—Figure 5. The seed cakes from pumpkin and sunflower are at the ends of the range—pumpkin with the lowest and sunflower with the highest O/C and H/C atomic ratio, respectively. This large dispersion is due to the complex composition of the seed cakes, especially in terms of proteins and types of lipid.

The torrefaction of seed cakes decreased the O/C and H/C atomic ratios to 0.18–0.20 and 0.63–0.81, respectively. This shifts the solid materials to the lower left side of the Van Krevelen diagram, closer to its origin, indicating their improved quality for use as solid fuels. Sunflower and walnuts had the strongest variation, which can be correlated to the highest liquid and gas yields, corresponding to the production of large amounts of water and carbon dioxide, respectively. The torrefied solid materials are less dispersed, showing a more uniform ratio between carbon, hydrogen, and oxygen. As mentioned before in Section 3.1, the main difference in thermal behaviour between the seed cakes comes from their complex compositions of extractives, mainly the proteins and fatty acids. It appears that torrefaction successfully removed large amounts of these components, flattening the composition of the solid materials. This is of high importance when torrefaction is used as a preliminary pre-treatment step before further valorisation via pyrolysis.

### 3.3. Composition of Organic Compounds in Liquid Products

The torrefaction of biomass produces mainly oxygen-containing compounds, since the C–O bonds induce weak points in the structure—unless they are adjacent to functional groups such as aromatic rings or alternating C=C double bonds—that are able to stabilize oxygen radicals by resonance. This usually leads to interactions between components and to the complex composition of the liquid products. Most chemical compounds of thermal degradation have significant polarity, hence they can partition into the aqueous and the oil phase. Therefore, the liquid product was vigorously shaken before GC-MSD analysis. The complex composition of liquid products can be easily visualized using NP-grams, which represent the distribution of compounds versus the carbon number of normal paraffins leaving the GC column in a similar retention time range [49].

**Figure 6:**
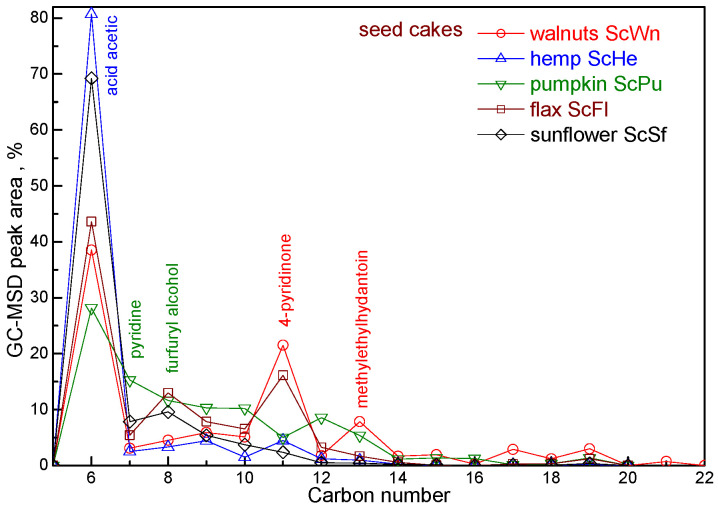
The NP-grams of the liquid products from the torrefaction of seed cakes.

**Figure 7:**
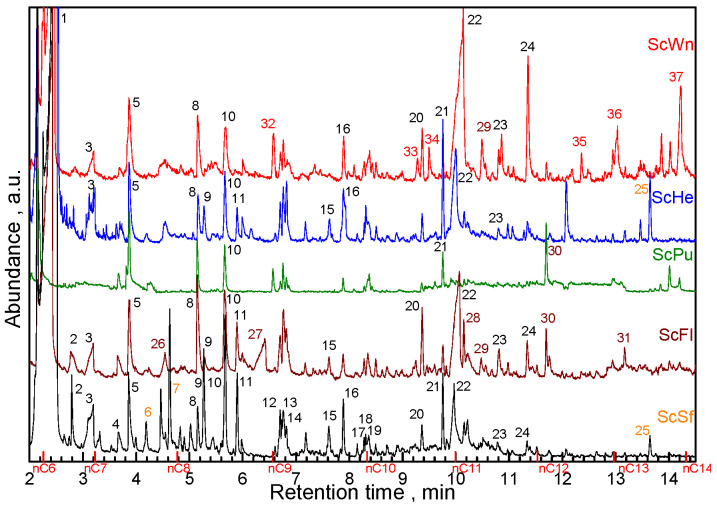
The GC-MS chromatograms of the liquid products from the torrefaction of seed cakes.

Most of the compounds in the liquid products (above 90%) are distributed in the nC6–nC14 range—with only small amounts being above nC14; these values are slightly higher for the seed cake from walnuts—Figure 6. Hence, the gas chromatograms in Figure 7 are presented only in the range below retention times of 14.5 min, corresponding to nC14.

The main peak is presented at nC6 and corresponds to acetic acid, which is a product from the thermal degradation of hemicelluloses—but also of lignin—especially the acetylated ones, as in herbaceous biomass [50]. This represents ~70 and 80% for sunflower and hemp (it should be mentioned that sunflower has the highest content of hemicelluloses and lignin) and between ~30 and 45% for the other three seed cakes (flax, walnut, and pumpkin, in decreasing order). A rather uniform distribution of compounds is observed between nC7 and nC10, with a small peak at nC8 for flax and sunflower, with furfuryl alcohol being the main compound in this range. A clear peak appears at nC11 for walnut and flax (21.5 and 16.0%, respectively), coming mainly from 4-pyridinone, which is only present in small amounts for the torrefaction liquid products from pumpkin, hemp, and sunflower. The composition of liquid products from the torrefaction of seed cakes can generally be described as more uniform for pumpkin but very unbalanced (predominantly acetic acid) for hemp.

The detailed composition of the liquid products from the torrefaction of seed cakes can be observed from the chromatograms in Figure 7, with the peak assessment listed in Table 1.

A total of 22 compounds were commonly found in the liquid products from all studied seed cakes; 6 compounds were particular to flax, and 6 were specific to walnuts. In addition to the 22 common compounds shared by all seed cakes, pumpkin has only one compound in common with flax (no. 30), and hemp has only one compound in common with sunflower (no. 25). However, the ratio between the compounds strongly differs between samples, as can be seen from the peak size in the chromatograms.

In addition to acetic acid, the main chemical compounds produced by the torrefaction of seed cakes—several hydroxy ketones (acetol and acetol acetate) and furan derivatives (furfural, furfuryl alcohol, and 5-methylfurfural)—come from the thermal degradation of hemicelluloses, while phenol and its methoxy-derivatives (guaiacol, syringol, and vinylguaiacol) are typical products of lignin structures. These are the general compounds that result from the torrefaction of vegetal biomass [51].

Most of the other compounds belong to the class of single- or double-nitrogen atom aromatic compounds—pyridine, pyrrole, and pyrazine. Several of them—for example, pyrrole, 2-acetylpyrrole, and indole, with the latter being formed from the thermal scission of tryptophan—were also reported by other researchers [52]. These are the thermal degradation products of the proteins present in seed cakes, which can be affected by interactions with the structural components. The corresponding oxygenated structures (succinimide and glutarimide) were also identified, especially in flax and walnuts. The nitrogen-containing compounds are particular related to seed cakes; these were not found in relevant amounts in the torrefaction liquids from other biomass residues from agriculture or fruit crops activities, such as stalks, hulls, or shells, or from forestry residues. This is due to the complex protein content of the seed cakes. Small amounts of free fatty acids still remaining in the cakes after cold press extraction appear at higher retention times (e.g., ~21 min. for palmitic acid and ~22 min. for linoleic acid) in the chromatograms of the torrefaction liquids.

**Figure 8:**
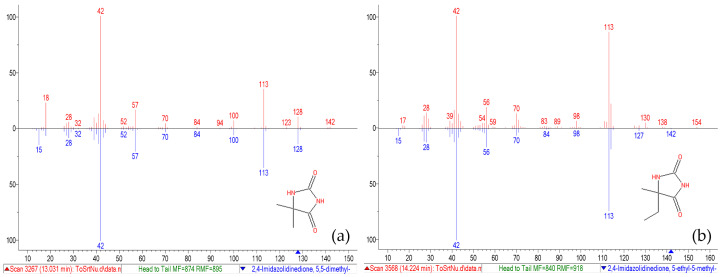
The MS spectrum of the GC peak (upper side, in red) and of the corresponding database compound (lower side, in blue) for hydantoin derivatives at 13.030 min (**a**) and 14.224 min (**b**).

Two compounds were found in significant amounts (1.94 and 3.20%) in the torrefaction liquids from walnuts only, but not for the other seed cakes. These are the dimethyl- and ethylmethyl-derivatives of 2,4-imidazolidinedione (hydantnoin), respectively. Their qualitative identification was based on a very good relative match (895 and 918) and probability (76.5 and 80%) of the spectrum with the database—Figure 8. While natural hydantoins were observed mainly in fungi and marine microorganisms [53], only a few papers report on their occurrence in plants, for example in horseradish [54]. Hydantoin-based compounds are largely used as fungicides, including in walnut cultures [55]. However, it is improbable for the high amounts of hydantoin derivatives identified in the torrefaction liquids of walnut seed cakes to originate from the possible treatments applied during walnut cultivation. Studies have also reported on the formation of ethylmethylhydantoin pyrolysis compounds derived from the poly-L-alanine peptide [56], or on hydantoins as pyrolysates of glycine- and arginine-free amino acids [57]. On the other hand, the production of hydantoin by heating allantoin in the presence of hydroiodic acid was reported by other studies [58,59]. As an analogy, it might be considered that iodine, which is present in high amounts in walnuts only but not in the other seeds, generates reactive species at the high temperature of torrefaction and these species can act as reducing agents, which interfere with the thermal degradation of amino acids, leading to hydantoin derivatives. While elucidating this mechanism is far beyond the purpose of this work, it might be a subject of interest for future studies.

## 4. Conclusions

Seed cakes from walnuts, hemp, pumpkin, flax, and sunflower were studied from the point of view of thermal stability and thermal valorisation. Thermogravimetry revealed that the seed cakes have a mass loss of about 66–70 wt% in a large temperature range of between 160 and 520 °C. The decomposition pattern is very complex, with numerous DTG shoulders corresponding to the volatilisation of fatty acids and the degradation of proteins. The torrefaction of seed cakes was performed at 250 °C in a semi-batch glass reactor, yielding 75–82 wt% solids, 14–20 wt% liquids, and 3–9 wt% non-condensable gasses. The torrefied solids have an energy density of ~1.2 and an energy yield of 91–97%, proving that torrefaction is a highly energy efficient process. This is due to the strong removal of oxygen, especially from physically and chemically bonded water. As a consequence, the H/C and especially the O/C atomic ratio decreased, moving the seed cakes towards the origin of the Van Krevelen diagram and closer to the range of superior solid fuels. The composition of liquid products is distributed mainly in the range of nC6–nC14 of the NP-grams, with the main peak at nC6 represented by acetic acid. Furans, hydroxy ketones, and phenols were the typical chemical compounds from hemicelluloses, cellulose, and lignin, and the interactions between them. Additionally, significant amounts of nitrogen-containing aromatic compounds from the classes of pyridine, pyrrole, pyrazine, and their derivatives were formed from the thermal degradation of protein constituents, which represent a major part of the seed cakes. Torrefaction was proven to be a valuable treatment/valorisation method to convert seed cakes from walnuts, hemp, pumpkin, flax, and sunflower into valuable biochars or bio-oils.

## Figures and Tables

**Table 1:** The main compounds (GC-MSD area %) in torrefaction liquids from seed cakes.

No	Rt	Ri	Name	CAS	ScSf	ScFl	ScPu	ScHe	ScWn
1	2.507	626	acetic acid	64-19-7	57.93	32.34	14.73	72.52	34.93
2	2.794	655	acetol	116-09-6	1.54	1.73	-	-	-
3	3.194	697	propanoic acid	79-09-4	2.83	1.82	-	0.00	0.85
4	3.672	729	pyrazine	290-37-9	0.86	0.71	3.56	0.15	0.47
5	3.867	741	pyridine/pyrrole	110-86-1/109-97-7	1.67	2.12	16.84	1.36	3.65
6	4.189	762	1-hydroxy-2-butanone	5077-67-8	0.80	-	-	-	-
26	4.544	785	3-butenoic acid	625-38-7	-	1.32	-	-	-
7	4.63	791	2,3-butanediol	19132-06-0	3.74	-	-	-	-
8	5.157	822	methylpyrazine	109-08-0	0.83	2.92	9.75	0.71	1.93
9	5.269	828	furfural	98-01-1	1.93	0.97	-	0.65	-
10	5.671	850	furfuryl alcohol	98-00-0	2.42	3.90	10.61	1.19	2.09
11	5.900	863	acetol acetate	592-20-1	1.12	1.78	-	0.48	-
27	6.415	892	3-methylcrotonic acid	541-47-9	-	3.60	-	-	-
32	6.581	901	4-propylcyclohexylamine	102653-37-2	-		-	-	1.16
12	6.703	908	butyrolactone	96-48-0	0.66	1.28	1.46	0.50	0.64
13	6.766	911	2,6-dimethylpyrazine	108-50-9	0.81	1.69	4.12	0.70	0.84
14	6.819	914	ethylpyrazine	13925-00-3	0.61	2.18	1.58	0.90	1.07
15	7.618	960	5-methylfurfural	620-02-0	0.78	0.77		0.45	
16	7.892	975	phenol	108-95-2	0.86	0.87	2.36	1.33	0.95
17	8.278	997	2-ethyl-6-methylpyrazine	13925-03-6	0.24	0.50	0.88	0.21	0.44
18	8.309	999	3-methoxypyridine	7295-76-3	0.24	-	2.26	0.37	0.29
19	8.366	1002	2-ethyl-5-methylpyrazine	13925-03-6	0.49	0.88	2.52	0.41	1.14
33	9.283	1057	furaneol	3658-77-3	-	-	-	-	0.64
20	9.362	1062	2-acetylpyrrole	1072-83-9	0.60	1.33	1.47	0.29	0.99
34	9.501	1070	2-pyrrolidinone	616-45-5	-	-	-	-	0.92
21	9.760	1086	guaiacol	90-05-1	1.21	0.73	5.40	1.30	0.50
22	9.971	1099	4-pyridinone	NIST433504	2.75	7.12	0.72	3.67	13.58
28	10.153	1110	maltol	118-71-8	-	1.18	-	-	-
29	10.477	1131	succinimide	123-56-8	-	0.66	-	-	1.59
23	10.785	1151	6-methyl-3-pyridinol	1121-78-4	0.51	1.13	-	0.42	1.02
24	11.341	1187	glutarimide	1121-89-7	0.23	0.93	0.84	0.41	3.15
30	11.697	1211	5,6-dihydro-2,4,6-trimethyl-4h-1,3,5-dithiazine	NIST360364	-	0.98	10.26	-	-
35	12.950	1299	indole	120-72-9	-	-	-	-	0.79
25	13.647	1350	syringol	91-10-1	0.24	-	-	0.69	-
36	13.030	1304	5,5-dimethylhydantoin	77-71-4	-	-	-	-	1.94
31	13.172	1315	p-vinylguaiacol	7786-61-0	-	1.54	-	-	-
37	14.224	1393	5-ethyl-5-methylhydantoin	5394-36-5	-	-	-	-	3.20

Compounds in black are found in most of the samples while coloured compounds are found in only in the sample with the same colour (orange for ScSf, wine for ScFl, red for ScWn).

## Data Availability

The original contributions presented in the study are included in the article, further inquiries can be directed to the corresponding author.

## References

[1] Hanif M.A., Nadeem F., Tariq R., Rashid U., Hanif M.A., Nadeem F., Tariq R., Rashid U. (2022). Energy resources and utilization. Renewable and Alternative Energy Resources.

[2] Muscat A., de Olde E.M., Ripoll-Bosch R., Van Zanten H.H.E., Metze T.A.P., Termeer C.J.A.M., van Ittersum M.K., de Boer I.J.M. (2021). Principles, drivers and opportunities of a circular bioeconomy. Nat. Food.

[3] Rahman M., de Jiménez M.M., Gupta S.K. (2016). Designer oil crops. Breeding Oilseed Crops for Sustainable Production.

[4] Dobrescu M. (2021). Romanian Oilseeds Poised for Big Recovery. USDA GAIN Report. https://apps.fas.usda.gov/newgainapi/api/Report/DownloadReportByFileName?fileName=Romanian%20Oilseeds%20Poised%20for%20Big%20Recovery_Bucharest_Romania_05-23-2021.pdf.

[5] Popescu A. (2020). Oilseeds crops: Sunflower. Rape and soybean cultivated surface and production in Romania in the period 2010–2019 and forecast for 2020–2024 horizon. Sci. Pap. Ser. Manag. Econ. Eng. Agric. Rural. Dev..

[6] Zhang M., Wang O., Cai S., Zhao L., Zhao L. (2023). Composition, functional properties, health benefits and applications of oilseed proteins: A systematic review. Int. Food Res..

[7] Rakita S., Kokić B., Manoni M., Mazzoleni S., Lin P., Luciano A., Ottoboni M., Cheli F., Pinotti L. (2023). Cold-pressed oilseed cakes as alternative and sustainable feed ingredients: A review. Foods.

[8] Petraru A., Ursachi F., Amariei S. (2021). Nutritional characteristics assessment of sunflower seeds, oil and cake. Perspective of using sunflower oilcakes as a functional ingredient. Plants.

[9] Sims I.M., Monro J.A., Boland M., Moughan P.J. (2013). Fiber: Composition, Structures, and Functional Properties. Advances in Food and Nutrition Research.

[10] Popović S., Hromiš N., Šuput D., Bulut S., Romanić R., Lazić V., Ramadan M.F. (2020). Valorization of by-products from the production of pressed edible oils to produce biopolymer films. Cold Pressed Oils. Green Technology, Bioactive Compounds, Functionality, and Applications.

[11] Arrutia F., Binner E., Williams P., Waldron K.W. (2020). Oilseeds beyond oil: Press cakes and meals supplying global protein requirements. Trends Food Sci. Technol..

[12] Potin F., Goure E., Lubbers S., Husson F., Saurel R. (2022). Functional properties of hemp protein concentrate obtained by alkaline extraction and successive ultrafiltration and spray-drying. Int. J. Food Sci. Technol..

[13] Mendoza-Pérez R.J., Náthia-Neves G., Blanco B., Vela A.J., Caballero P.A., Ronda F. (2024). Physicochemical characterisation of seeds, oil and defatted cake of three hempseed varieties cultivated in Spain. Foods.

[14] Singh R., Sá A.G.A., Sharma S., Nadimi M., Paliwal J., House J.D., Koksel F. (2023). Effects of feed moisture content on the physical and nutritional quality attributes of sunflower meal-based high-moisture meat analogues. Food Bioprocess Technol..

[15] González-Pérez S., Vereijken J.M. (2007). Sunflower proteins: Overview of their physicochemical, structural and functional properties. J. Sci. Food. Agric..

[16] Mirpoor S.F., Giosafatto C.V.L., Porta R. (2021). Biorefining of seed oil cakes as industrial co-streams for production of innovative bioplastics. A Rev. Trends Food Sci. Technol..

[17] Ogunronbi O., Jooste P.J., Abu J.O., Van Der Merwe B. (2011). Chemical composition, storage stability and effect of cold-pressed flaxseed oil cake inclusion on bread quality. J. Food Process. Preserv..

[18] Zdunczyk Z., Minakowski D., Frejnagel S., Flis M. (1999). Comparative study of the chemical composition and nutritional value of pumpkin seed cake, soybean meal and casein. Mol. Nutr. Food Res..

[19] Sarkis J.R., Côrrea A.P.F., Michel I., Brandeli A., Tessaro I.C., Marczak L.D.F. (2014). Evaluation of the phenolic content and antioxidant activity of different seed and nut cakes from the edible oil industry. J. Am. Oil Chem. Soc..

[20] Pramanik J., Kumar A., Prajapati B. (2023). A review on flaxseeds: Nutritional profile, health benefits, value added products, and toxicity. eFood.

[21] Mattila P.H., Pihlava J.-M., Hellström J., Nurmi M., Eurola M., Mäkinen S., Jalava T., Pihlanto A. (2018). Contents of phytochemicals and antinutritional factors in commercial protein-rich plant products. Food Qual. Saf..

[22] Náthia-Neves G., Alonso E. (2021). Valorization of sunflower by-product using microwave-assisted extraction to obtain a rich protein flour: Recovery of chlorogenic acid, phenolic content and antioxidant capacity. Food Bioprod. Process..

[23] Wildermuth S.R., Young E.E., Were L.M. (2016). Chlorogenic acid oxidation and its reaction with sunflower proteins to form green-colored complexes. Compr. Rev. Food Sci. Food Saf..

[24] Lanier C., Heutte N., Richard E., Bouchart V., Lebailly P., Garon D. (2009). Mycoflora and mycotoxin production in oilseed cakes during farm storage. J. Agric. Food Chem..

[25] Sun Y., Li C., Li Q., Zhang S., Xu L., Gholizadeh M., Hu X. (2021). Pyrolysis of flaxseed residue: Exploration of characteristics of the biochar and bio-oil products. J. Energ. Inst..

[26] Wang Y., Zhao Y., Hu C. (2024). Slow pyrolysis of de-oiled rapeseed cake: Influence of pyrolysis parameters on the yield and characteristics of the liquid obtained. Energies.

[27] Rajpoot L., Tagade A., Deshpande G., Verma K., Geed S.R., Patle D.S., Sawarkar A.N. (2022). An overview of pyrolysis of de-oiled cakes for the production of biochar, bio-oil, and pyro-gas: Current status, challenges, and future perspective. Biores. Technol. Rep..

[28] Butnaru E., Pamfil D., Stoleru E., Brebu M. (2022). Characterization of bark, needles and cones from silver fir (*Abies alba* Mill.) towards valorization of biomass forestry residues. Biomass Bioenerg..

[29] Petrovič A., Predikaka T.C., Vohl S., Hostnik G., Finšgar M., Čuček L. (2024). Hydrothermal conversion of oilseed cakes into valuable products: Influence of operating conditions and whey as an alternative process liquid on product properties and their utilization. Energy Convers. Manag..

[30] Mishra R.K., Mohanty K. (2018). Pyrolysis kinetics and thermal behavior of waste sawdust biomass using thermogravimetric analysis. Biores. Technol..

[31] Butnaru E., Stoleru E., Brebu M. (2022). Valorization of forestry residues by thermal methods. The effect of temperature on gradual degradation of structural components in bark from silver fir (*Abies alba* Mill.). Ind. Crops. Prod..

[32] Butnaru E., Brebu M. (2022). Torrefaction process of needles, cones and bark of spruce (*Picea abies* (L.) Karst) and pine (*Pinus sylvestris* L.). Rev. Chim..

[33] Bárta J., Bártová V., Jarošová M., Švajner J., Smetana P., Kadlec J., Filip V., Kyselka J., Berčíková M., Zdráhal Z. (2021). Oilseed cake flour composition, functional properties and antioxidant potential as effects of sieving and species differences. Foods.

[34] Butnaru E., Brebu M. (2022). The thermochemical conversion of forestry residues from silver fir (*Abies alba* Mill.) by torrefaction and pyrolysis. Energies.

[35] Junges J., Silvestre W.P., De Conto D., Baldasso C., Osório E., Godinho M. (2020). Non-isothermal kinetic study of fodder radish seed cake pyrolysis: Performance of model-free and model-fitting methods. Braz. J. Chem. Eng..

[36] Silvestre W.P., Pauletti G.F., Baldasso C. (2020). Fodder radish (*Raphanus sativus* L.) seed cake as a feedstock for pyrolysis. Ind. Crop. Prod..

[37] Shen L., Alexander K.S. (1999). A thermal analysis study of long chain fatty acids. Thermochim. Acta.

[38] Melzera M., Blina J., Bensakhriac A., Valetteb J., Broust F. (2013). Pyrolysis of extractive rich agroindustrial residues. J. Anal. Appl. Pyrolysis.

[39] Smets K., Schreurs S., Carleer R., Yperman J. (2014). Valorization of raspberry seed cake by flash and slow pyrolysis: Product yield and characterization of the liquid and solid fraction. J. Anal. Appl. Pyrolysis.

[40] Stedile T., Ender L., Meier H.F., Simionatto E.L., Wiggers V.R. (2015). Comparison between physical properties and chemical composition of bio-oils derived from lignocellulose and triglyceride sources. Renew. Sustain. Energy Rev..

[41] Bach Q.V., Chen W.H. (2017). A comprehensive study on pyrolysis kinetics of microalgal biomass. Energy Convers. Manag..

[42] Li H., Niu S., Lu C. (2017). Thermal characteristics and kinetic calculation of castor oil pyrolysis. Procedia Eng..

[43] Li X., Mei Q., Dai X., Ding G. (2017). Effect of anaerobic digestion on sequential pyrolysis kinetics of organic solid wastes using thermogravimetric analysis and distributed activation energy model. Bioresour. Technol..

[44] Wei X., Ma X., Peng X., Yao Z., Yang F., Dai M. (2018). Comparative investigation between co-pyrolysis characteristics of protein and carbohydrate by TG-FTIR and Py-GC/MS. J. Anal. Appl. Pyrolysis.

[45] Braga R.M., Costa T.R., Freitas J.C.O., Barros J.M.F., Melo D.M.A., Melo M.A.F. (2014). Pyrolysis kinetics of elephant grass pretreated biomasses. J. Therm. Anal. Calorim..

[46] Liu G., Wright M.M., Zhao Q., Brown R.C., Wang K., Xue Y. (2016). Catalytic pyrolysis of amino acids: Comparison of aliphatic amino acid and cyclic amino acid. Energy Convers. Manag..

[47] Mitan N.M.M., Brebu M., Bhaskar T., Muto A., Sakata Y., Kaji M. (2007). Co-processing of DVDs and CDs with vegetable cooking oil by thermal degradation. J. Mater. Cycles Waste Manag..

[48] Channiwala S.A., Parikh P.P. (2002). A unified correlation for estimating HHV of solid, liquid and gaseous fuels. Fuel.

[49] Murata K., Makino T. (1975). Thermal degradation of polypropylene. Nippon Kagaku Kaishi.

[50] Zhou S., Xue Y., Sharma A., Bai X. (2016). Valorization through thermochemical conversion: Comparison of hardwood, softwood and herbaceous lignin. ACS Sustain. Chem. Eng..

[51] Vasile C., Brebu M.A. (2006). Thermal valorisation of biomass and of synthetic polymer waste. Upgrading of pyrolysis oils. Cell. Chem. Technol..

[52] Ucar S., Ozkan A.R. (2008). Characterization of products from the pyrolysis of rapeseed oil cake. Bioresour. Technol..

[53] Youssef D.T., Shaala L.A., Alshali K.Z. (2015). Bioactive hydantoin alkaloids from the red sea marine sponge *Hemimycale arabica*. Mar. Drugs.

[54] Lee T.H., Khan Z., Kim S.Y., Lee K.R. (2019). Thiohydantoin and hydantoin derivatives from the roots of *Armoracia rusticana* and their neurotrophic and anti-neuroinflammatory activities. J. Nat. Prod..

[55] Liu X., Yang K.Q., Zhu Y.F., Yin Y.F. (2013). In vitro virulence of eight fungicides to *Colletotrichum gloeosporioides* of walnut anthracnose. Chin. J. Pestic. Sci..

[56] Basiuk V.A., Douda J. (2000). Pyrolysis of poly-glycine and poly-l-alanine: Analysis of less-volatile products by gas chromatography/Fourier transform infrared spectroscopy/mass spectrometry. J. Anal. Appl. Pyrol..

[57] Moldoveanu S.C., Moldoveanu S.C. (2019). Pyrolysis of amino acids and small peptides. Pyrolysis of Organic Molecules.

[58] Hugh C. (1911). Hydantoin. Encyclopædia Britannica.

[59] Terzuoli L., Pizzichini M., Arezzini L., Pandolfi M.L., Marinello E., Pagani R. (1994). Separation and determination of allantoin, uric acid, hydantoin and urea. J. Chromatogr. A.

